# Association between Physical Activity and Health Outcomes (High Body Fatness, High Blood Pressure) in Namibian Adolescents and Adult women

**DOI:** 10.3390/ijerph21040446

**Published:** 2024-04-05

**Authors:** Hilde Liisa Nashandi, Andries Makama Monyeki, John J. Reilly

**Affiliations:** 1 School of Nursing and Public Health, Faculty of Health Sciences and Veterinary Medicines, University of Namibia, Windhoek 10005, Namibia; 2 Physical Activity, Sport and Recreation Research Focus Area, Faculty of Health Sciences, North-West University, Potchefstroom 2520, South Africa; andries.monyeki@nwu.ac.za; 3 Department of Psychological Sciences and Health, University of Strathclyde, Glasgow G1 1QE, UK; john.j.reilly@strath.ac.uk

**Keywords:** physical activity, blood pressure, high body fatness, adolescent girls, adult women, anthropometry

## Abstract

Regular physical activity (PA) is known to promote the physical and mental health of children and adolescents and further prevent the development of health problems in adulthood. Information on body composition and PA is crucial for health promotion strategies and for epidemiological studies informing policies. However, there is limited data on the association between body composition and PA in Namibia. This dearth of published data is a significant shortcoming in the development of strategies and policies to promote PA in Namibia. Therefore, this cross-sectional study was conducted to determine the association between PA as a dependent variable and independent variables such as high blood pressure and body fatness as measured by different methods (gold standard deuterium dilution, body mass index, mid upper arm circumference, and waist circumference). The study included 206 healthy adolescent girls aged 13–19 years and 207 young adult females aged 20–40 years from Windhoek, Namibia. PA was measured using the PACE+ questionnaire in adolescents, and the GPAQ questionnaire was used for adults. In adolescents, only 33% of the participants met the recommended guidelines for PA, compared to only 2% for adults. Nevertheless, the study found no statistically significant association between PA and blood pressure indices (*p*-value < 0.05) among adolescents and adults. However, there was a significant association between PA and high body fatness (*p*-value < 0.001) and waist circumference (*p*-value = 0.014) in adolescents. Among adults, PA was significantly related to waist circumference only. In conclusion, failure to meet recommended PA guidelines is strongly associated with abdominal obesity and high body fatness. The knowledge gained from this study may be used by policymakers in the development of strategic policies and interventions aimed at promoting PA as a public priority and improving health outcomes.

## 1. Introduction

Global progress to improve PA levels to the recommended World Health Organisation (WHO) guidelines has been slow, largely due to the lack of awareness of PA benefits and financial investment challenges [1]. The WHO report on Global Action for PA (2018) states that 1 in 4 adults and 3 in 4 adolescents (aged 11 to 17) do not meet the global recommendations for PA set by WHO (2020), which call for adults to engage in at least 75 to 150 min of vigorous intensity aerobic physical activity per week or 60 min or more of moderate-to-vigorous physical activity (MVPA) per day for children and adolescents and 150 to 300 min of moderate-intensity aerobic physical activity for adults [1,2]. The survey also states that up to 70% of individuals live physically sedentary lifestyles in some nations [2]. According to a review by Aubert et al. (2021) [3], children and adolescents worldwide do not have enough levels of PA.

According to the Active Healthy Kids Global Alliance (AHKGA), no more than one-third of children and adolescents worldwide are estimated to satisfy the WHO (2020) guideline [4] (p. 200). Furthermore, only 20% of in-school adolescents met the WHO’s recommended PA level, according to a multi-country analysis of Global School-Based Health Surveys from 23 African countries, including Namibia. The Survey also showed that teenage girls in Africa are less active than teenage boys [5].

According to the Namibia Demography Health Survey (NDHS) conducted in 2013, only 5% of females and 12% of men aged 15–49 are physically active when they are at work, while 16% of females and 32% of men engage in non-work-related PA (meaning being physically active during their free time when they are not at work) [6]. According to the latest 2013 Namibia Global School-Based Student Health Survey (GSHS) [7], 10.4 percent of female students aged 13–17 years were overweight or obese. The NDHS (2013) in Namibia reported that urban females (40% prevalence) are more likely to be overweight or obese compared to rural females (22% prevalence), but the last NDHS was published in 2013, and that is the only national data available. A study performed in Namibia among NAMDEB mine employees found a prevalence of 42% being overweight and 32% being obese [8].

Globally, childhood and adult obesity figures are soaring, with girls and females being mostly affected [9] (pp. 2627–2642), with a rapid increase in developing countries like those in Sub-Saharan Africa. It has been observed that a higher body mass index (overweight and obesity) is linked to hypertension in children, adolescents, and adults [10]. Chen et al. (2023) [11] found that about 10% of adolescents in sub-Saharan Africa between the ages of 10 and 19 have high blood pressure. High blood pressure is a significant concern because of its rising prevalence. In Namibia, as per the latest Namibia Demographic Health Survey (2013) [6], 44% of women between the ages of 35 and 64 had high blood pressure (BP). Numerous studies have demonstrated that high blood pressure during childhood and adolescence is a critical element in the development of hypertension in adulthood. High blood pressure is a recognised primary risk factor for stroke and coronary heart disease [11,12].

A study by Tian and Zhang (2022) [13] in China found that there was a statistically significant relationship between PA and high BP in adults aged 50 years and older. Hence, research suggests that a high level of PA may lead to a low risk of high blood pressure [13,14]. A review by Valenzuela et al. (2021) [15] revealed strong evidence that supports the benefits of regular PA and exercise for the prevention and management of hypertension. A study by You et al. (2020) among pre-university students in Malaysia concluded that there was a significant association between PA and body composition. Meanwhile, the same study found no significant relationship between PA and BMI [16]. A systematic review by Kraus et al. (2018) highlighted that adults must reach the recommended PA guidelines to reduce premature mortality and cardiovascular disease (CVD) risk [17]. Namibia GSHS (2013) [7] found that less than 15% of adolescents met the previous WHO guidelines for MVPA. An epidemiological study focusing on the association between PA and hypertension as well as the association between PA and body fat mass as measured by various methods, such as gold standard deuterium dilution, body mass index (BMI), mid upper arm circumference (MUAC), and waist circumference, is warranted because data on the association between body composition and PA in Namibia could not be found. This study focuses on girls and women since they are more physically inactive compared to boys and men.

## 2. Methods

### 2.1. Study Setting and Participants

This study is part of a larger study called the TC project (NAM6011/2018) on “Determining risk factors of obesity and non-communicable diet-related diseases (NCDs) in adolescent girls, and adult female in Windhoek, Khomas region, Namibia”. The study was conducted as a cross-sectional study aimed to determine body composition, PA, dietary habits, nutritional status, and selected lifestyle health risk factors in Namibian adolescent girls and young adult females. The study had 206 adolescent girls from four (4) public schools; at least 50 adolescents per school participated. These adolescents were chosen randomly from school registers and were 13–19 years of age.

A total of 207 young women, between the ages of 20 and 40, who worked as teachers and staff in 18 public schools were selected at random to participate in the study as adults. Each school included a minimum of 11 adult female participants in the research. The inclusion criteria took into account healthy adult females (aged 20–40) and healthy girls (aged 13–19). Exclusion criteria were applied to subjects with complications associated with large shifts in body water (water retention or dehydration); pregnant women; and any other physical or mental condition that might impair the subject’s ability to participate in the study. The parents of the adolescents who took part in the study signed consent forms on behalf of their adolescents, while the adults signed their own consent [18].

Data collection and all measurements took place at schools for all the participants. For the purpose of this study, the data that was collected in 2018 was used to determine the association between PA and health outcomes (e.g., high body fatness, high blood pressure) in adolescents and adult females from the Khomas region, Windhoek, Namibia.

### 2.2. Ethics

The Ethical Research Committee of the Ministry of Health and Social Services (MOHSS) in Namibia examined and approved the study protocol (reference#: 17/3/3/MV), as did the North-West University Health Research Ethics Committee (NWU-HREC) with ethics number (NWU–00017–23–A1). Permission was also granted by the Executive Director of the Ministry of Basic Education, Arts, and Culture before the start of the data collection process. All adults give informed consent on behalf of participating adolescents, and those who did not wish their children to participate were free to opt out without any negative consequences. Adolescents further signed an assent form. Adult female participants signed a written informed consent form to participate in the study. Strict confidentiality was ensured, and the data were only accessible to those involved in the study.

### 2.3. Body Composition Measurements

#### 2.3.1. Body Fatness Measurement by the Deuterium Dilution Method in Adolescents and Adults

Measurements of total body water by deuterium through saliva were used to determine body composition (body fatness) [19]. Adults’ body fat percentage was confirmed to be accurately calculated using this reference technique [20]. Fat-free mass (FFM) is computed using the predicted total body weight (TBW). The difference between FFM and body weight is called body fat mass (FM) [19]. The subjects were given an oral dose of deuterium oxide after the baseline saliva sample was taken, and two and three hours later, post-dose saliva samples were taken to allow for equilibration with body water compartments for the computation of TBW. 

The enrichment of deuterium was measured using a portable FTIR (Agilent 4500 Series Tumble-IR portable FTIR spectrometer). This machine is manufactured by Agilent Technologies Australia [M] Pty, Ltd. in Mulgrave, Australia. This equipment is known to be more robust and sensitive than conventional transmission FTIRs with a liquid cell [19]. FTIR instrumentation is easier to use and maintain, is less expensive to buy, and the cost of analysis is lower. It is therefore particularly suitable in resource-limited settings. 

Adolescents’ hydration of fat-free mass is decreasing due to their continuing growth [19,21]. For this reason, TBW-derived body fat mass measurements were produced by using established age- and sex-specific constants for the hydration of fat-free mass [19]. According to reports, the water content range for adolescents’ FFM was 74.5–75.5% [19]. Since growth has ceased and a constant for the hydration of fat-free mass is appropriate, the standard value of 73.2% for the hydration of fat-free mass was used to quantify fat mass from TBW in adults [18]. The difference between body weight and FFM relative to body weight was used to compute BF and BF%.

In this study, for adolescents, a >30% cutoff for body fatness has been used to indicate high body fatness, associated with marked increases in cardio-metabolic risk factors [22]. This cutoff for high body fatness has been widely used elsewhere in studies of the classification accuracy of BMI, as summarised in previous systematic reviews [23,24]. 

In adults, a 38% cutoff for body fatness has been used to indicate overfatness. This increase in body fatness is associated with higher risk markers for chronic diseases/conditions in black females [25] and has been used previously in different studies, such as [18,26], to support the concept that this cutoff is fairly widely used.

#### 2.3.2. Weight and Height Measurements for BMI Calculation

Using conventional procedures and methodologies, two trained researchers measured weight (in light indoor clothing) to 0.1 kg and height to 0.1 cm in adults and adolescents [27]. A child/adult/infant portable height-length measuring board was used as a height gauge. Height is measured barefoot. The participant puts their heels on the board while standing straight up. The head is positioned on Frankfurt’s plane. Keeping their knees straight, the subjects are asked to look directly ahead. The eyes and ears being at the same level must be verified. 

The participant’s weight was measured to the nearest 0.1 kg using an electronic SECA scale with adequate precision. The balance was placed on a level surface. Participants wear minimal clothing, with items removed from their pockets, and no shoes. An accurate measurement of body weight is essential. The accuracy of the scale was checked regularly using a calibration weight of a known mass.

BMI was calculated as per the formula of weight/height^2^ (kg/m^2^). As recommended by the WHO, an adult BMI of >25 kg/m^2^ defined as overweight, and a BMI of >30 kg/m^2^ is defined as obese in adults. These values were used to analyse weight status in adults as recommended by the WHO. Adolescent BMI categories for adolescent participants were assigned based upon BMI-for-age z-scores calculated using the WHO Child Growth Standards (2006) [28]. WHO reference tables (2006), categorised as obesity (BMI-for-age > +2SD), overweight (BMI-for-age > +1SD), and undernutrition/wasting (BMI-for-age < −2SD).

#### 2.3.3. Mid Upper Arm Circumference (MUAC) Measurement

After the arm was flexed to a 90-degree angle from the elbow, MUAC was measured on the left arm using non-stretchable plastic tape at the midpoint between the olecranon and the acromial process. The measurement was taken to the closest 0.1 cm [18], the arm was relaxed, and the MUAC tape was wrapped around the arm’s designated midpoint without being excessively tight or too loose. The literature provides evidence of the MUAC’s satisfactory validity in screening for obesity and overweight [29]. 

In the present study, the MUAC cut-off of 22.6 cm was used as a proxy definition of obesity because it has been used to indicate excessive fatness in adolescent females from South Africa [30,31]. Both of the MUAC cut-offs of 30.6 cm and 33.0 cm were used to characterise obesity in the adult participants in this study since they have been suggested in Southern Africa for identifying obesity in women presenting for obstetric care at antenatal care booking visits in early pregnancy [32,33].

#### 2.3.4. Waist Circumference Measurement

The study participants were standing and breathing normally when the waist circumference was measured, with a Lufkin non-stretch measuring tape, at the midpoint between the last rib and the top of the iliac crest (hip bone), to the nearest 0.1 cm.

Waist circumference was used alone as a measure of risk, as it is an indicator of abdominal obesity, which is a risk factor for cardio-metabolic conditions and diseases (CMD). Waist circumference as an outcome measure has been validated for assessing visceral fat, % body fat, and visceral fat changes over time [34]. Adults’ various cutoffs from the World Health Organisation and International Diabetes Federation (−80.0 and 88.0 cm) were used in this study as proxies for obesity; additionally, a higher cut-off of 91.5 cm was taken from a study that examined the associations between waist circumference and cardiometabolic risk factors in South African adult women [18,35]. For adolescents, a cut-off point for waist circumference of 80.0 cm was selected [18]. 

#### 2.3.5. Measurement of Physical Activity (PA)

Participants were interviewed using validated questionnaires that have some evidence of validity from other populations and/or are being used in Namibia in national surveillance, whereby the findings show the effectiveness of the implemented interventions [36]. For adolescents, the PACE+ questionnaire was used, and for adults, the GPAQ questionnaire was used. Study participants are asked to describe how many days in the previous week they spent engaging in MVPA for at least 60 min using the PACE+ questionnaire (examples of activities are provided). Participants provided the following outcomes: VPA during free time (days and hours per week); daily transportation to and from school; walking time to school (hours per day); TV watching time (hours per day); and computer or phone game playing (hours per day). After adding up all of the answers to each question, a total of 86 scores were obtained; individuals scoring less than 43 were classified as having low PA, while those scoring more than 43 were classified as having high PA.

It has been determined that GPAQ is a suitable measure for evaluating the efficacy of initiatives aimed at enhancing MVPA [37]. 

As part of the WHO STEPS NCDs surveillance project, GPAQ is extensively used worldwide to monitor adult compliance with physical activity levels and also serves as a sitting time measure [38]. When the total scores from all the questions were added up, it was determined that people with half of these scores, or less than 23, had high PA, and people with more than 23, had low PA. 

By summing up different activities, the PACE+ and GPAQ assessments were used to determine an individual’s level of PA and classify them as meeting or not meeting the physical activity guidelines.

#### 2.3.6. Measurement of Blood Pressure

Resting blood pressure was measured with a digital automatic OMRON blood pressure monitor while the participant was sitting. Three readings of systolic and diastolic blood pressure were taken on the right arm using the appropriate cuff size for each participant after a 15 min rest. The participant rests for three minutes between each of the readings. During data analysis, the mean of the second and third readings was calculated to be used for analyses. The blood pressure cut-offs for adolescents and adults are the same and are categorised as follows: Normal BP is defined as systolic BP (SBP) < 120 mmHg and diastolic BP (DBP) < 80 mmHg; raised BP is defined as SBP of 120–129 mmHg and DBP < 80 mmHg; hypertension stage 1 is SBP 130–139 mmHg and/or DBP 80–89 mmHg, and hypertension stage 2 is SBP ≥ 140 mmHg and/or DBP ≥ 90 mmHg [39,40].

### 2.4. Data Analysis

The statistical analysis was performed using the SPSS 27.0 software program version 27 (IBM Corp., Armonk, NY, USA, 2020). A separate analysis was performed for adolescents and adults. For continuous variables, means and standard deviations were calculated, while for categorical variables, frequencies and percentages were analysed. Physical activity was categorised into high and low levels of PA. The level of PA was determined as follows as per their age categories: high PA is when individual physical activities meet the recommended WHO PA guidelines, low PA is when individual physical activities do not meet the recommended WHO PA guidelines. Associations between PA and blood pressure, waist circumference, MUAC, BMI, and body fatness by TBW were analysed using the chi-squared and Fisher’s exact tests whenever applicable.

## 3. Results

### 3.1. Characteristics of Study Participants

Only 206 adolescents and 207 adult females provided the required information used for statistical analysis. Table 1 depicts the sample characteristics. 

### 3.2. Physical Activity in Adolescents

As indicated in Table 2, 67 (33%) adolescents met the PA guidelines as recommended by the WHO and were classified as having high PA based on the PACE+ questionnaire. Further results of the PACE+ are shown in Supplementary Table S1. 

Table 2 shows the association between physical activity and BMI-z-score, mid-upper arm circumference, blood pressure, body fat percentage, and waist circumference among adolescents. In addition, Table 2 shows the prevalence of obesity as determined by different measures of body composition. Table 2 shows that not meeting PA guidelines is significantly associated with high body fatness by TBW (*p*-value < 0.001) and abdominal obesity by waist circumference (*p*-value = 0.014). On average, an adolescent who did not meet PA guidelines has a high chance (72.7%) of having high body fatness, whereas an adolescent who met PA guidelines has a high chance (63.3%) of having normal body fatness. Alternatively, on average, an adolescent who did not meet the PA guidelines is two times more likely to have high body fatness compared to those who met the PA guidelines (73%/37%). Similarly, on average, an adolescent who did not meet PA guidelines is more likely (83.3%) to have abdominal obesity compared to those with a normal WC (63.4%). 

This table also shows that there was no statistically significant association between physical activity and blood pressure (*p* = 0.89), PA and MUAC (*p* = 0.91), or PA and BMI (*p* = 0.37). 

### 3.3. Physical Activity in Adults

This study showed that only 4/207 (1.9%) of adults had PA levels as recommended by WHO guidelines, while the majority 203/207 (98.1%) did not meet the PA guidelines. 

Table 3 shows that not meeting PA guidelines is significantly associated with abdominal obesity as measured by waist circumference (*p*-value = 0.027) in adults. On average, an adult who did not meet PA guidelines is more likely (100%) to have abdominal obesity by WC compared to those with a normal waist circumference (96.5%). As indicated above, there was no statistically significant association between physical activity and blood pressure. The prevalence of not meeting PA guidelines among adult participants with high BP was 97%, while the prevalence of not meeting recommended PA guidelines among adult participants with normal BP was 98%. The table also indicates that there was no statistically significant association between PA and other variables of health outcomes because the *p*-value was >0.05, such as PA and MUAC (*p* = 0.18), PA and body fatness (*p* = 0.33), and PA and BMI (*p* = 0.32). Supplementary Table S2 further depicts the descriptive results of the GPAQ for adults.

## 4. Discussion

### 4.1. Main Findings and Study Implications

This study found that 33% of the adolescents and 2% of the adults meet the WHO PA guidelines. This is a worrisome finding of a decline in PA in Namibia. This study among adolescents and adult women found that there is no significant association between physical activity and blood pressure (*p*-value = 0.89 > 0.05), and this finding is similar to the study performed by Papathanasiou et al. (2015) [41], which also revealed that PA status was not associated with BP in a sample of Greek young adults. The results show that not meeting PA guidelines is associated with a high chance of adolescents and adults being abdominally obese by waist circumference. This is consistent with Okely et al. (2019) [42], which stipulate that higher levels of PA may be associated with healthy weight status in adolescents. Abdominal obesity is the main cause of CVD and mortality worldwide. There is a significant association between PA and high body fatness, whereby adolescents who meet PA guidelines turn out to have normal body fatness, as the *p* = value is 0.001 (<0.05). 

These findings motivate the researchers/authors to recommend interventions that help adolescents be physically active and meet the recommended PA guidelines, which have positive health outcomes and curb obesity in adulthood. Because of the increased autonomy during this phase and the potential for positive behaviours developed during this time to persist into later adulthood, behaviour change therapies should focus on the adolescence–adulthood transition.

The results show no evidence of a statistically significant association between PA and obesity among adolescents, as measured by MUAC and BMI for age z score. These findings necessitate repeat future studies with robust methodological designs and a large sample size to re-examine this relationship in the Namibian context. 

### 4.2. Comparison with Other Literature Studies

As it appears in this study, it is a known fact that physical activity is lower in adulthood than in adolescence [43]. Although in this study on one side, there was no statistically significant relationship between PA and BP, MUAC, BMI, or body fatness by TBW, these findings can be due to physical inactivity among adults as found in other studies [44,45]. The significant association between PA and waist circumference in adults relates to the same results of [45]. Abdominal obesity as measured by WC is a stronger predictor of cardiovascular diseases, metabolic syndrome, morbidity, and mortality as expressed by Armstrong et al. (2022) [46] and Ref. [47]; therefore, high-intensity exercises are required to reduce WC. It is emphasized in many studies that PA has many benefits for the health of individuals. 

The present study findings contrast with the findings by Gamage and Seneviratne (2021) [48] and Ref. [49], which identified physical inactivity as an important independent contributor to hypertension. Syaugi (2021) discovered that physical activity lowers blood pressure, particularly systolic blood pressure. It is recommended that, in addition to focusing on increasing physical activity, people also make dietary changes in order to maintain normal blood pressure [50].

Arija et al. (2018) indicated that a PA intervention programme improved cardiovascular health and health related quality of life and favoured blood pressure control in participants with hypertension [51]. Other studies by Bakker et al. (2018) [52]; Ref. [53] agree that promoting physical activity and improving fitness, especially (cardiorespiratory fitness), should be encouraged in order to lower the risk of hypertension and decrease blood pressure. Gibbs et al. (2021) stipulated that increasing PA has extensive benefits for body composition and blood pressure [54].

### 4.3. Strengths of the Study

The current study was the first in Namibia to explore the association between PA and blood pressure as well as the association between PA and adiposity as measured by the deuterium dilution technique, which is a gold standard, and proxies BMI, MUAC, and waist circumference among adolescents and adult women. There is a significant and strong association between PA, waist circumference, and high body fatness among adolescents. A significant association was also found between PA and waist circumference in adults. 

When administered over time, the PACE+ and GPAQ questionnaires may yield valuable information about population levels of physical activity and trends among Namibian adult and adolescent females. These findings support the need for intervention research to examine and contrast the causal association between various markers of adiposity in populations and physical activity.

### 4.4. Weakness of the Study

Despite the above-mentioned strengths, the present study had limitations that were important to consider when interpreting the results. The self-reporting of physical activity in the study poses limitations. This could lead to an overreporting of physical activity because of a bias brought about by desire. Recall bias is another issue with self-reporting that can cause physical activity scores to be overestimated or underestimated. As this study included only women in urban areas, the results may not be confidently applied to adolescent boys and adult men and also may not be generalized to those in rural areas. In addition, our present study findings should be interpreted with caution because of the small sample size of the female adolescents and adults recruited in the study relative to the entire population of female adolescents and adults in Windhoek. Another limitation of the study is its cross-sectional nature, which does not allow for causal inferences between the dependent and independent variables in the study. A larger study with a larger sample size, including men and women from both rural and urban areas, is required. Future studies can also include confounding variables that may influence the health outcome, which were not the focus of this study.

## 5. Conclusions

This study concludes that PA levels are low in both adolescents and adult women, although the prevalence is higher in adults than adolescents. PA is significantly associated with waist circumference and high body fatness in adults and adolescents’ females in Windhoek urban areas. 

It is a known fact that a decrease in physical activity can harm health in the short term during childhood and adolescence, as well as in the long term during adulthood. Abdominal obesity and high body fatness among adolescents and adults can be prevented by putting more emphasis on PA as an intervention.

## Figures and Tables

**Table 1:** Characteristics of study participants, mean (SD).

Variable	Adolescents (*n* = 206)	Adults (*n* = 207)
Age (y)	16.5 (1.6)	31.5 (5.8)
Grade * n (%)		
8	33 (16.0)	-
9	43 (20.9)	-
10	61 (29.6)	-
11	27 (13.1)	-
12	42 (20.4)	-
Occupation n (%)		
Yes	-	157 (75.8)
No	-	50 (24.1)
Educational level n (%)		
Primary school	-	3 (1.4)
Secondary school	-	47 (22.7)
College/University	-	137 (17.9)
Postgraduate	-	9 (4.3)
Refused to answer	-	16 (7.7)
Socio-economic status n (%)		
Low-density suburbs	-	131 (63.3)
Middle-density suburbs	-	64 (30.9)
High-density suburb	-	11 (5.3)
Height (cm)	159.9 (5.9)	161.7 (5.9)
Weight (kg)	56.9 (13.6)	71.2 (17.0)
BMI (kg/m^2^)	22.2 (4.9)	27.1 (6.3)
BMI Z-score	0.22 (1.28)	Not applicable
MUAC (cm)	26.2 (4.0)	30.5 (4.6)
Waist Circumference (cm)	72.9 (11.0)	87.1 (14.4)
SBP (mmHg)	114.8 (13.9)	120.1 (14.9)
DBP (mmHg)	72.4 (10.5)	78.8 (11.6)
TBW (kg)	27.7 (4.0)	31.0 (4.6)
Fat Mass (kg)	19.8 (9.5)	28.8 (12.0)
Fat-Free Mass (kg)	37.1 (5.5)	42.4 (6.2)
Body fat (% of body weight) from TBW	33.4 (7.9)	34.0 (7.7)

SBP = systolic blood pressure, DBP = diastolic blood pressure, TBW = total body water, MUAC = mid upper arm circumference, BMI = body mass index, * Grade represents the grade at school the child is at the time of the study, e.g., Grade 8 is the first year in secondary school and Grade 12 is the last year in secondary school. Place of residence was used as a proxy for socio-economic status and was divided into high, medium, and low-density suburbs.

**Table 2:** Crosstabulation between physical activity and BMI-z-score, MUAC, blood pressure, body fat percentage, and waist circumference among adolescents.

Variables	Physical Activity (row %)		Total	Pearson $\chi^{2}$ Statistic, d.f. and *p*-Value
	Low	High		
BMI Z Score				
obese	16 (76.2)	5 (23.8)	21 (100)	0.809, 1, 0.368
normal	123 (66.5)	62 (33.5)	185 (100)	
Total	139	67	206	
MUAC				
obese	117 (67.6)	56 (32.4)	173 (100)	0.012, 1, 0.914
normal	22 (66.7)	11 (33.3)	33 (100)	
Total	139	67	206	
Blood pressure				
high blood pressure	47 (68.1)	22 (31.9)	69 (100)	0.019, 1, 0.889
normal	92 (67.2)	45 (32.8)	137 (100)	
Total	139	67	206	
Body fatness				
high body fatness	128 (72.7)	48 (27.3)	176 (100)	15.187, 1, <0.001
normal	11 (36.7)	19 (63.3)	30 (100)	
Total	139	67	206	
Waist circumference				
abdominal obesity	35 (83.3)	7 (16.7)	42 (100)	6.045, 1, 0.014
normal	104 (63.4)	60 (36.6)	164 (100)	
Total	139	67	206	

**Table 3:** Crosstabulation between physical activity and BMI, MUAC, blood pressure, body fat percentage, and waist circumference among adult women.

Variables	Physical Activity (row %)		Total	*p*-Value (Fisher’s Exact)
	Low	High		
BMI				0.32
obese	61 (100)	0 (0)	61 (100)	
normal	142 (97.3)	4 (2.7)	146 (100)	
Total	203	4	207	
MUAC				0.32
obese	63 (100)	0 (0)	63 (100)	
normal	140 (97.2)	4 (2.8)	144 (100)	
Total	203	4	207	
Blood Pressure				0.63
high BP	110 (97.3)	3 (2.7)	113 (100)	
normal	93 (98.9)	1 (1.1)	94 (100)	
Total	203	4	207	
Body Fatness				0.32
high body fatness	146 (98.6)	2 (1.4)	148 (100)	
normal	57 (96.6)	2 (3.4)	59 (100)	
Total	203	4	207	
Wait circumference				0.027
abdominal obesity	94 (100)	0 (0)	94 (100)	
normal	109 (96.5)	4 (3.5)	113 (100)	
Total	203	4	207	

## Data Availability

The data presented in this study are available on request from the corresponding author.

## Supplementary Materials

The following supporting information can be downloaded at: https://www.mdpi.com/article/10.3390/ijerph21040446/s1, Table S1: PACE+ variables and descriptive information; Table S2: GPAQ variables and descriptive information.
