# Supplementary material for: Association between Physical Activity and Health Outcomes (High Body Fatness, High Blood Pressure) in Namibian Adolescents and Adult women
Source: Int J Environ Res Public Health. 2024 Apr 5;21(4):446. doi: 10.3390/ijerph21040446 (PMC11050568; doi:10.3390/ijerph21040446)
Supplement: Supplementary file 1 [file ijerph-21-00446-s001.zip › ijerph-2855418-supplementary.pdf]

Supplementary Table S1: PACE+ variables and descriptive information

PACE+ variables	Responses	Yes, n (%)
How long does it usually take you to travel to school from your home?	Less than 5 minutes<br>5-15 minutes<br>15-30 minutes<br>30 minutes to 1 hour<br>More than 1 hour	62(30.1)<br>0 (0.0)<br>10(4.9)<br>68(33.0)<br>66(32.0)
How many hours a day, in your free time, do you usually spend watching TV, videos (including YouTube or similar services), DVDs, and other entertainment on a screen? Weekdays / Weekend.	None at all<br>About half an hour a day<br>About 1 hour a day<br>About 2 hours a day<br>About 3 hours a day<br>About 4 hours a day<br>About 5 hours a day<br>About 6 hours a day<br>About 7 or more hours a day)	12(5.8)<br>17(8.3)<br>18 (8.7)<br>34 (16.5)<br>46 (22.3)<br>30 (14.6)<br>22 (10.7)<br>11 (5.3)<br>16 (7.7)
How many hours a day, in your free time, do you usually spend playing games on a computer, games console, tablet (like iPad), smartphone or other electronic device (not including moving or fitness games)? Weekdays / Weekend	None at all<br>About half an hour a day<br>About 1 hour a day<br>About 2 hours a day<br>About 3 hours a day<br>About 4 hours a day<br>About 5 hours a day<br>About 6 hours a day<br>About 7 or more hours a day)	86 (41.7)<br>38 (18.4)<br>32 (15.5)<br>21 (10.2)<br>12 (5.8)<br>9 (4.4)<br>5 (2.4)<br>1 (0.5)<br>2(1.0)

Supplementary Table S2: GPAQ variables and descriptive information

GPAQ variables	Yes, n (%)	No, n (%)	Mean (SD)
Does your work involve vigorous-intensity activity that causes large increases in breathing or heart rate like [carrying or lifting heavy loads, digging or construction work] for at least 10 minutes continuously	3(1.4)	204 (98.6)	N/A
In a typical week, on how many days do you do vigorous-intensity activities as part of your work? (days/week)	N/A	N/A	3(2.0)
How much time do you spend doing vigorous-intensity activities at work on a typical day? (hours/day)	N/A	N/A	1.7(1.2)
Does your work involve moderate-intensity activity, that causes small increases in breathing or heart rate such as brisk walking [or carrying light loads] for at least 10 minutes continuously?	29(14.0)	178(86.0)	N/A
In a typical week, on how many days do you do moderate-intensity activities as part of your work? (days/week)	N/A	N/A	2.8(2.0)
How much time do you spend doing moderate-intensity activities at work on a typical day? (hours/day)	N/A	N/A	2.5 (2.6)
Do you walk or use a bicycle (pedal cycle) for at least 10 minutes continuously to get to and from places?	113 (54.6)	94(45.4)	N/A
In a typical week, on how many days do you walk or bicycle for at least 10 minutes continuously to get to and from places? (days/week)	N/A	N/A	4.4 (2.0)
How much time do you spend walking or bicycling for travel on a typical day? (hours/day)	N/A	N/A	0.5 (0.8)
